# Patterns of treatment for psychiatric disorders among children and adolescents in Mississippi Medicaid

**DOI:** 10.1371/journal.pone.0221251

**Published:** 2019-08-15

**Authors:** John Young, Sujith Ramachandran, Andrew J. Freeman, John P. Bentley, Benjamin F. Banahan

**Affiliations:** 1 Department of Psychology, University of Mississippi, Oxford, MS, United States of America; 2 Department of Pharmacy Administration, University of Mississippi, Oxford, MS, United States of America; 3 Department of Psychology, University of Nevada, Las Vegas, NV, United States of America; University of Tennessee Health Science Center, UNITED STATES

## Abstract

The nature of services for psychiatric disorders in public health systems has been understudied, particularly with regard to frequency, duration, and costs. The current study examines patterns of service reception and costs among Medicaid-covered youth newly diagnosed with anxiety, depression, or behavioral disturbance in a large data set of provider billing claims submitted between 2015–2016. Eligibility criteria included: 1) identification of an initial diagnosis of a single anxiety, unipolar mood, or specific behavioral disorder; 2) continuous Medicaid eligibility over the duration of the time period studied; and 3) under 18 years of age on the date of initial psychiatric diagnosis. The final cohort included 7,627 cases with a mean age of 10.65 (±4.36), of which 58.04% were male, 57.09% were Black, 38.97% were White, and 3.95% were of other ethnicities. Data indicated that 65.94% of the cohort received at least some follow-up services within a median 18 days of diagnosis. Of those, 54.27% received a combination of medical and psychosocial services, 32.01% received medical services only, and 13.72% received psychosocial services only. Overall median costs for direct treatment were $576.69, with wide discrepancies between the lowest (anxiety = $308.41) and highest (behavioral disturbance = $653.59) diagnostic categories. Across all categories the frequency and duration of psychosocial services were much lower than would be expected in comparison to data from a well-known effectiveness trial. Overall, follow-up to psychiatric diagnosis could be characterized as highly variable, underutilized, and emphasizing biomedical treatment. Understanding more about these patterns may facilitate systematic improvements and greater cost efficiency in the future.

## Introduction

Mental health disorders are prevalent in children and adolescents [1–4] and often exhibit a chronic course without intervention [5–7]. Youth who experience psychopathology have more negative life outcomes such as poor school performance, involvement with child welfare or juvenile justice systems, and illicit substance use than typically developing youth [8]. Unfortunately, fewer than 1 in 4 afflicted youth receive services [9], potentially at least partly due to mental health treatment being stigmatized [9,10] and limited accessibility (particularly in terms of evidence-based treatments [7,11–21]). The mismatch between those in need and those receiving services is unfortunate given that numerous psychosocial treatments for youth have accumulated empirical support in a wide range of service settings [22–27].

Explanations for why youth fail to intersect with appropriate services are complex, multifaceted, and presently unclear. Economic factors, shifting policies toward mental health parity, expansion of Medicaid coverage, and adaptation to the Affordable Care Act (ACA [28,29]) have all increased the potential for adequate mental healthcare for youth. The provision of evidence-supported mental healthcare, however, appears to be both limited in prevalence and duration and becoming less common over time [30–32]. Despite widely disseminated practice guidelines calling for psychosocial interventions as primary interventions, the majority of clinicians report infrequent use of evidence-supported treatment strategies [33–37]. Instead, prescription medication as the sole treatment approach for psychiatric symptoms has been increasing, even in contexts where evidence for these approaches is mixed or lacking entirely [38, 39].

The reasons for this trend in service provision are not clear, and previous studies using cross-sectional, random samples of the national patient population to examine the issue do not provide elucidation [31]. This methodology provides insight into generalities about the US mental health system overall, but potentially ignores evidence that healthcare utilization patterns differ substantially on the basis of ethnicity, geographic location, and other social factors [32,40–42]. More localized, longitudinal examinations of the types, patterns, and costs of services received for psychiatric conditions would advance contemporary understanding in this domain.

The current study utilizes secondary data from the Mississippi Division of Medicaid, which included all child and adolescent billing claims submitted during a contiguous two-year period. Specifically, longitudinal service patterns related to identifiable psychiatric diagnoses were examined and broadly categorized as medical office visits only, psychosocial treatment only, or combined treatment. The primary purpose of the current manuscript was to understand more about the typical form of service provision, including provider type, time between meetings, treatment duration, and costs.

## Methods

### Data source

Data were sourced from all Mississippi Medicaid claims submitted between January 1, 2015 and December 31, 2016. Databases examined included files detailing beneficiary demographics, provider information, and all pharmacy, outpatient, medical, and inpatient claims submitted for billing. All statistical analyses were conducted with SAS 9.4 (SAS Institute, Cary, NC). The project was approved by the University of Mississippi Institutional Review Board and the data were accessed after approval from the Mississippi Division of Medicaid Privacy Board.

### Inclusion & exclusion criteria

Study participants were required to be continuously eligible in Mississippi Medicaid for the duration of the study period. Beneficiaries were included in the data set if they were newly diagnosed with a psychiatric disorder from one of the following categories: anxiety, unipolar depression, or a specific behavioral disturbance (i.e., conduct disorder or oppositional defiant disorder). These general categories were utilized to allow direct comparisons of the patterns of psychosocial service utilization seen in the current data to the MacArthur Child STEPs project (a well-known randomized clinical effectiveness trial [25] that included details about the frequency and timing of psychosocial treatments). Specific ICD codes for diagnoses comprising each category appear in the S1 Appendix.

All participants’ records were identified using ICD-9 or -10 diagnostic codes present in the outpatient, inpatient, or medical claims file (Table 1) between July 1^st^, 2015 and December 31^st^, 2015. An index date (i.e., date of initial diagnosis) was established within this time frame for all cases meeting inclusionary criteria. Diagnoses were verified as new by examining data from January 1, 2015 through June 30, 2015 to ensure that no earlier claims included reference to the diagnosis of interest. In instances where the identified diagnosis was present between January and June, 2015, the case was excluded from the study. Similarly, any indication of psychiatric comorbidity during this 6-month lookback period led to exclusion. Finally, beneficiaries were also excluded if they were 18 years of age or older on the index date. All cases retained for study thus included at least 12 months of continuous data between the index date and the end of the range represented in the data (December 31^st^, 2016).

**Table 1:** Demographic and diagnostic profile on index date.

	Diagnosis			
	Anxiety	Depression	Behavioral Disturbance	Overall
Total *n* (%)	1888 (24.75)	1088 (14.27)	4651 (60.98)	7627
Female *n* (%)	1028 (54.45)	687 (63.14)	1485 (31.93)	3200 (41.96)
Age *M* (SD)	9.60 (5.10)	14.13 (2.77)	10.27 (3.92)	10.65 (4.36)
Race/Ethnicity *n* (%)				
Black	794 (42.06)	561 (51.56)	2999 (64.48)	4354 (57.09)
White	987 (52.28)	484 (44.49)	1501 (32.27)	2972 (38.97)
Other	107 (5.67)	43 (3.95)	151 (3.25)	301 (3.95)

Although these inclusionary criteria are somewhat restrictive, they allow for a detailed view of services rendered for a focal problem type. There is also previous evidence to suggest that intake assessments in the course of community-based clinical services result in assignment of a single diagnosis [43]. The procedures employed were thus likely to result in cases with diagnostic profiles similar to what would be encountered in other public healthcare systems when considering these problem types.

### Outcome measures

The frequency of follow-up medical office visits and psychosocial service visits, time to initial follow up, duration of treatment, specialties of the diagnosing and treating providers, and costs for services were recorded for all beneficiaries in the final data set. These were compiled by first identifying all claims for a given individual that included the relevant psychiatric diagnostic code. Among this set, those that contained CPT codes identifying a psychosocial service (90832, 90834, 90837, 90846, 90847, 90791, 90853, 96101, 96111, 90618, H2012, 90785, 96127, 96110) were defined as psychosocial visits. Any claims beyond the index date that referenced the initial psychiatric diagnosis but did not contain one or more of these CPTs were labeled as follow-up medical office visits.

All study participants were classified as receiving either follow-up medical office visits only, psychosocial services only, both medical office visits and psychosocial services (i.e., combined treatment), or neither (i.e., the diagnosis of interest did not appear after the index date). For any beneficiaries receiving services beyond the initial diagnosis, the time between the index date and the next identified service (regardless of type) was defined as the time to initial follow up. Duration of treatment was defined as the time between the index date and the *last* follow-up medical office visit or psychosocial service during the study period.

Provider specialties were obtained from the National Provider Identifier (NPI) number associated with each claim. The self-reported nature of NPIs produced a diverse array of data describing clinical specialties, some of which referred to institutions and some to individuals. For ease of interpretation, NPI numbers were therefore classified as belonging to one of the following categories: General Medical Clinic or Hospital (GMCH), Inpatient Hospitalization (INPT), Mental Health Practitioner (MHPR), Non-Mental Health Practitioner (NMPR), Nurse Practitioner or Physician Assistant (NPPA), Psychologist (PSYO), Psychiatrist (PSYR), Social Services (SOCS), Occupational Therapist (OCTR), Other (OTHR), or unknown (UNKW).

The beneficiary demographics file was used to record the beneficiary’s age on the index date, gender, and ethnicity. Data for costs of follow-up treatment were compiled using Mississippi Medicaid’s reimbursement rate for each CPT code for the appropriate fiscal year, which yielded specific expenditures for services. It should be noted, however, that these calculations did not include the cost of prescription drugs (given that it was impossible to connect medications to a specific diagnosis or medical office visit). The economic data that follow are thus representative of direct provider service costs *only*.

Finally, the patterns of service reception for each diagnostic category were compared to those noted in the MacArthur Child STEPs treatment effectiveness study [25], which indicated that individuals randomly assigned to manualized treatments received a mean of 16.17 (±9.95) sessions separated by an average of 11.96 (±4.65) days. Given that this trial demonstrated superior outcomes for manualized approaches delivered in applied settings, these figures provide a convenient means of comparing real-world patterns of service utilization to what might reasonably be expected if evidentiary services were being provided. The current data set was thus examined in terms of the frequency of individuals who 1) received a number of psychosocial sessions within one standard deviation of the MacArthur mean; 2) exhibited a pattern of timing between sessions within one standard deviation of the MacArthur mean; and 3) met both of these criteria.

## Results

### Participants

The selection process resulted in a cohort of 7,627 individuals who were predominantly male (58.04%) with a mean age of 10.65 (±4.36). Ethnic composition of this group was as follows: 57.09% Black (n = 4,354); 38.97% White (n = 2,972); 3.95% Other (n = 301). Table 1 reports diagnoses by demographic categories, which were significantly different as a function of both ethnicity (χ^2^ (4) = 292.86; p <0.0001) and gender (χ^2^ (2) = 513.59; p < 0.0001).

### Initial diagnosis

As seen in Table 2, the most common provider type associated with initial psychiatric diagnosis was MHPR (31.18%), which included counselors and clinical social workers (but not psychologists or psychiatrists, each of which had its own category). The next-most frequent provider type was NMPR, which primarily comprised physicians with a specialty other than psychiatry (22.62%). Considered in combination with the frequencies attributable to GMHCs (16.06%) or NPPAs (6.99%), it appears that roughly half of initial diagnoses were rendered in non-specialized medical settings. Alternatively, diagnosis by a psychologist (6.08%) or psychiatrist (2.05%) was fairly rare. The rates of provider types also differed by diagnostic category, with medical/hospital facilities more likely to be the source of initial anxiety diagnoses and mental health practitioners more likely to provide diagnoses of depression and behavioral disturbance.

**Table 2:** Diagnosis and treatment provider type.

Provider Type–Initial Diagnosis											
**Diagnosis**	**Total n**	**GMCH**	**INPT**	**NMPR**	**NPPA**	**OCTR** **& OTHR**	**PSYO**	**PSYR**	**SOCS**	**MHPR**	**UNKW**
Anxiety n (%)	1886	679 (36.00)	6 (0.32)	533 (28.26)	182 (9.65)	31 (1.64)	58 (3.08)	31 (1.64)	69 (3.66)	151 (8.01)	146 (7.74)
Depression n (%)	1086	142 (13.08)	56 (5.16)	194 (17.86)	98 (9.02)	21 (1.93)	79 (7.27)	42 (3.87)	44 (4.05)	331 (30.48)	79 (7.27)
Behavioral Disorder n (%)	4641	402 (8.66)	220 (4.74)	995 (21.44)	252 (5.43)	179 (3.86)	326 (7.02)	83 (1.79)	166 (3.58)	1892 (40.77)	126 (2.71)
Overall n (%)	7613	1223 (16.06)	282 (3.70)	1722 (22.62)	532 (6.99)	231 (3.04)	463 (6.08)	156 (2.05)	279 (3.66)	2374 (31.18)	351 (4.61)
Provider Type–Follow-Up Treatment											
**Diagnosis**	**Total n**	**GMCH**	**INPT**	**NMPR**	**NPPA**	**OCTR** **& OTHR**	**PSYO**	**PSYR**	**SOCS**	**MHPR**	**UNKW**
Anxiety n (%)	688	81 (11.77)	3 (0.44)	158 (22.97)	82 (11.92)	21 (3.05)	54 (7.85)	31 (4.51)	59 (8.58)	141 (20.49)	58 (8.43)
Depression n (%)	769	46 (5.98)	14 (1.82)	91 (11.83)	36 (4.68)	16 (2.08)	73 (9.49)	81 (10.53)	33 (4.29)	302 (39.27)	77 (10.01)
Behavioral Disorder n (%)	3564	132 (3.70)	54 (1.52)	601 (16.86)	100 (2.81)	202 (5.67)	228 (6.40)	196 (5.50)	180 (5.05)	1757 (49.30)	114 (3.20)
Overall n (%)	5021	259 (5.16)	71 (1.41)	850 (16.93)	218 (4.34)	239 (4.76)	355 (7.07)	308 (6.13)	272 (5.42)	2200 (43.82)	249 (4.96)

Abbreviations: GMCH, General Medical Clinic or Hospital; INPT, Inpatient Hospitalization; NMPR, Non-Mental Health Practitioner; NPPA, Nurse Practitioner or Physician Assistant; OCTR, Occupational Therapist; OTHR, Other; PSYO, Psychologist; PSYR, Psychiatrist, SOCS, Social Services; MHPR, Mental Health Practitioner; UNKW, Unknown

*Note*: The group sizes in this table do not correspond to the overall number of participants due to provider NPI being missing from some billing claims. These cases were separated from the “unknown” category given qualitative differences in these groups (i.e., unable to discern professional specialty from existing NPI and provider self-description vs. information entirely absent).

### Follow-up services

Approximately two-thirds (65.94%) of beneficiaries received services beyond their initial diagnosis (Table 3). Among these, the median time to first follow-up visit was 18 days, although the amount of delay was variable depending on diagnostic category (Table 4). Youth with depression or behavioral disturbances were seen fairly quickly with median delays of 14 and 16 days (respectively), but the same statistic for youth with anxiety was 30 days. These times varied substantially, however, as a function of the type of follow-up services, with longer delays notable in groups that received medical office visits as their sole form of treatment. Additionally, when examining the rates of follow-up by age group it was apparent that children age 6 and younger received additional services significantly less often (40.1%) than those ages 7–12 (70.3%) and 13–17 (73%).

**Table 3:** Number of youth receiving follow-up services by diagnosis and service type.

Diagnosis	Medical Office Only n (%)	Psychosocial Only n (%)	Medical Office & Psychosocial n (%)	Neither n (%)
Anxiety	371 (19.65)	115 (6.09)	204 (10.81)	1198 (63.45)
Depression	236 (21.69)	116 (10.66)	417 (38.33)	319 (29.32)
Behavioral Disorder	1003 (21.57)	459 (9.87)	2108 (45.32)	1081 (23.24)
Overall	1610 (21.11)	690 (9.05)	2729 (35.78)	2598 (34.06)

*Note*: Percentages are row percentages.

**Table 4:** Duration in days to initial follow-up visit among those receiving any services after diagnosis.

	Overall			Medical Office Only			Psychosocial Only			Medical Office & Psychosocial		
Diagnosis	Mean (SD)	Median	n	Mean (SD)	Median	n	Mean (SD)	Median	n	Mean (SD)	Median	n
Anxiety	88.83 (121.23)	30	690	123.16 (137.61)	66	371	36.50 (60.60)	21	115	55.89 (92.26)	15	204
Depression	51.25 (89.79)	14	769	93.43 (123.23)	34	236	41.11 (70.35)	17.5	116	30.20 (58.80)	11	417
Behavioral Disorder	49.82 (82.83)	16	3570	90.28 (110.43)	38	1003	43.15 (73.78)	38	459	32.03 (59.44)	11	2108
Overall	55.39 (91.07)	18	5029	98.32 (119.80)	42	1610	41.70 (71.12)	16	690	33.53 (62.69)	11	2729

Among those receiving follow-up services, youth were less likely to receive follow-up visits through GMCH (5.16%) or NMPR (16.93%) in comparison to initial diagnoses. Psychologists (7.07%), psychiatrists (6.13%), and other mental health practitioners (43.82%), however, represented a larger percentage of follow-up than initial visits, perhaps reflective of referral to these professionals by less specialized providers. Similar to the data for initial diagnoses, there was also variability in the percentage of provider types across categories (see Table 2).

Of the youth who received follow-up services (n = 5,029), more than half received a combination of medical office and psychosocial services (54.27%), one-third received medical office visits only (32.01%), and the remainder received psychosocial services as their sole means of treatment (13.72%). Youth with anxiety were least likely to receive any follow-up services (36.55%), whereas the frequency of youth with depression (70.68%) or behavioral disturbances (76.76%) receiving at least some treatment was approximately twice as high. Finally, youth receiving combined treatment registered more psychosocial visits (18.39±29.70) than those receiving psychosocial services alone (8.18±16.55).

Comparison of the group receiving any follow-up services to those who received none indicated significant differences in several demographic categories. Specifically, the no follow-up group had a larger percentage of females (46.8% vs. 39.4%) different distribution of ethnicities (Black: 49.8% vs. 60.9%; White: 45.6% vs. 35.6%; Other: 4.7% vs. 3.6%), and was younger (mean 9.4 years of age ±4.8 vs 11.32±3.9; all *p*s < 0.0001). Additionally, these groups differed significantly in terms of the type of provider assigning the original diagnosis. The most common type for the no follow-up group was non-mental health practitioners (35.2%), whereas in the group that received subsequent services mental health practitioners were the most frequent (43.0%).

### Service costs

Costs of treatment were examined using all charges associated with any billing claim beyond the initial diagnosis that included the psychiatric diagnosis of interest. The median cost of treatment per individual receiving any follow-up services was $576.69. This amount differed substantially by category from a low of $308.41 for anxiety to a high of $653.59 for behavioral disturbances. Dividing these costs further by diagnostic category and provider type, it was apparent that combined treatment was consistently more costly (i.e., between 200–600% higher within-category median expenditures than either of the other treatment types; see Table 5).

**Table 5:** Direct service costs by category for cases receiving services beyond initial diagnosis (*excluding* pharmacy costs).

Average Cost Per Non-Zero Beneficiary in US Dollars									
Diagnosis	Number of Beneficiaries	Overall Cost		Medical Office Only		Psychosocial Only		Medical Office & Psychosocial	
		Mean (SD)	Median	Mean (SD)	Median	Mean (SD)	Median	Mean (SD)	Median
Anxiety	636	733.44 (1257.18)	308.41	432.37 (960.39)	180.80	560.36 (799.28)	274.16	1346.72 (1638.08)	762.18
Depression	727	1372.02 (3244.52)	580.61	680.39 (3507.85)	247.22	668.25 (1271.20)	265.73	1906.74 (3337.38)	1140.87
Behavioral Disorder	3450	2342.91 (7650.04)	653.59	2056.44 (11613.33)	231.72	749.72 (1572.80)	266.46	2801.55 (5933.51)	1335.57
Overall	4813	1983.58 (6640.70)	576.69	1487.78 (9336.02)	219.56	706.17 (1428.89)	266.04	2559.37 (5420.46)	1233.07

*Note*: The number of beneficiaries in this table does not correspond to the total number receiving services beyond initial diagnosis in Table 3, given that a number of individuals had follow-up services that were billed to Medicaid but not reimbursed. The data in this table represent *only* those cases for which reimbursement occurred (in an attempt to present typical capitated expenditures by the payer system for a given condition, as opposed to providers’ realized payments).

### Patterns of utilization

Among individuals receiving any psychosocial follow-up services, 1,187 received a total number of sessions within one standard deviation of the MacArthur mean (34.71% of the group receiving follow-up psychosocial services). A similar examination for the amount of elapsed time between sessions indicated that 83 individuals were within one standard deviation of the MacArthur mean (2.43%). Only 14 cases met both criteria when examining their patterns of service utilization (0.41%). For additional details about these patterns as a function of diagnostic category, please see Tables 6 and 7.

**Table 6:** Number of psychosocial services rendered.

Diagnosis	Overall Mean (SD)	Psychosocial Only Mean (SD)	Medical Office & Psychosocial Mean (SD)
Anxiety (n = 319)	4.83 (12.40)	6.71 (11.66)	12.54 (18.46)
Depression (n = 533)	8.28 (26.97)	7.74 (17.51)	13.11 (17.82)
Behavioral Disorder (n = 2567)	12.92 (26.97)	8.65 (17.33)	20.00 (32.15)
Overall (n = 3419)	11.10 (24.19)	8.18 (16.55)	18.39 (29.70)

**Table 7:** Duration of psychosocial services in weeks.

Diagnosis	Overall Mean (SD)	Psychosocial Only Mean (SD)	Medical Office & Psychosocial Mean (SD)
Anxiety (n = 319)	33.60 (23.48)	21.77 (20.67)	40.26 (22.35)
Depression (n = 533)	37.51 (22.89)	22.63 (20.60)	41.65 (21.77)
Behavioral Disorder (n = 2567)	35.91 (23.25)	19.77 (19.18)	39.42 (22.57)
Overall (n = 3419)	35.95 (23.22)	20.59 (19.68)	39.83 (22.44)

## Discussion

The current study examined patterns of psychiatric diagnosis and treatment in a large cohort of state-level child and adolescent Medicaid claims data over a two-year period. Approximately two-thirds of individuals with a formal mental health diagnosis received some follow-up services, which exceeded commonly cited base rates in the literature (between approximately 12–50% [44,45]). Initial diagnoses appear to have been rendered primarily in medical environments, but follow-up treatment occurred disproportionately in settings that were more focused on treatment of psychiatric symptoms. Although the reasons for this observation are not clear, one possibility is that diagnosing providers referred youth to specialized providers (consistent with the intended framework of an integrated system of care). Regardless, it was also apparent that psychosocial services were not typically emphasized as front-line treatments, despite strong evidence for the effectiveness of these techniques. The reasons for this outcome were also unclear, although it is similar to other national administrative claims research and a trend toward intervention through medication. One possible explanation is that the limited density of psychosocial providers in a primarily rural, underserved state made it very difficult to find and regularly receive services. Similarly, family financial limitations could have resulted in additional difficulties. The likelihood of both of these issues simultaneously being present was also very high, which could have resulted in effectively impassable barriers to receiving appropriate psychosocial care. In turn, such conditions could have facilitated decisions to pursue more widely available psychotropic interventions (independent of family preferences).

Additionally, it was notable that patterns of service utilization were extremely variable as a function of both diagnostic category and type of services received. For example, the rate of individuals seeking treatment for anxiety was approximately half that of the other diagnoses, and their delay in initial follow-up was nearly twice as long. Similarly, psychosocial services were both more likely and more frequent for behavioral disturbance, and the frequency of medical consultation was highest for cases of depression. Although these data may appear chaotic in overview, general trends did emerge (which could potentially facilitate the application of similar techniques to other administrative claims databases).

Independent of trends in service provision, data on economic expenditures suggested wide discrepancies depending upon the category of services received. Unsurprisingly, combined treatment was typically substantially more costly than psychosocial or medical office visits only. These services were also provided over a longer period of time with limited indication that they more closely matched patterns likely to be consistent with evidentiary treatment. More generally, resources expended for treatment across all categories (psychosocial or otherwise) were limited on a capitated basis. Given the possibility of exponentially escalating healthcare costs associated with psychiatric diagnosis over the course of an individual’s lifetime, under-treating current symptoms could result in much higher expenditures in the future. At the same time, data on current costs for psychosocial treatment can also be conceptualized as infinitely high, given that the patterns of services observed were inconsistent with evidentiary treatment in comparison to a known standard derived from work conducted in applied settings. The return on investment for these resources in terms of promoting individual health, longevity, productivity, or adjustment is therefore potentially minimal, and the long-term effectiveness of the system paying for these services is not enhanced as a result.

Future assessment of individual decision-making surrounding psychiatric diagnosis and treatment could provide greater insights, which in turn could lead to better adaptations of evidence-supported treatments to a wide range of settings. In particular, exploration of longitudinal administrative claims data or other large data repositories may shed light on trajectories of behavior subsequent to psychiatric diagnosis and discern predictors of specific paths (e.g., no treatment vs. pharmacotherapy vs. psychosocial treatment, etc.). Emphasis on individual, longitudinal, ethnographic research may also contribute to understanding how people orient to these problems and seek solutions for their healthcare needs, and how providers/healthcare service provision networks may optimize outcomes.

Observations such as those outlined above with regard to anxiety (made using passively collected administrative claims data and techniques that could easily be automated) could also be useful to facilitate service enhancement through the application of behavioral economic techniques [46,47]. Knowing that the base rate of treatment for symptoms of anxiety is low and the delay to follow-up is long could confer the opportunity to provide newly diagnosed families with behavioral nudges [48]. For example, indicating that 50% of people diagnosed with conditions other than anxiety who seek additional services tend to see a provider within two weeks could subtly encourage families to move more quickly. This simple, low-cost method could easily be randomized to enable administrative systems to learn more about their beneficiaries’ decision-making processes and the fiscal impact of encouraging pursuit of services.

Limitations of the current study should also be highlighted. First, youth were restrictively selected to represent first known identification of a single diagnosis, which likely resulted in discrepancies in the proportions of problem types (i.e., anxiety, depression, and disruptive behavior) compared to what might otherwise be expected for clinical settings. Likewise, this restrictive selection may limit generalizability to public health data when multiple comorbidities or recurrent episodes are considered. At the same time, this design strategy allowed a direct examination of the initial treatment episode and ensured limited confounds in mapping the reception of services over time. Additionally, the comparison of trends in service provision to a known standard in the Child STEPs study were coarse at best, albeit reflective of the best comparison data currently available. It is possible that patterns of services in the current data did not match those seen in STEPs due to positive treatment outcomes being achieved in much less time. It should be noted that this is unlikely, however, given limited previous evidence for positive outcome in treatment as usual [25,49,50]. Alternatively, there could be numerous environmental explanations that are independent of the quality of services provided (as outlined above). It was unfortunately not feasible to discern further details using administrative claims data, and more applied, organizational research will be necessary to understand these issues. Future studies combining “big data” approaches and primary research methods may address these limitations, advance integration of research and practice, and ultimately inform healthcare policy.

## Supporting information

S1 Appendix ICD-10 CM codes for diagnoses by category. (DOCX)
