# Supplementary material for: Patterns of treatment for psychiatric disorders among children and adolescents in Mississippi Medicaid
Source: PLoS One. 2019 Aug 15;14(8):e0221251. doi: 10.1371/journal.pone.0221251 (PMC6695227; doi:10.1371/journal.pone.0221251)
Supplement: S1 Appendix — (DOCX) [file pone.0221251.s001.docx]

S1 Appendix

ICD-10 CM codes for diagnoses by category

| Anxiety | Unipolar Depression | Behavioral Disturbance |
| --- | --- | --- |
| F40.0  F40.1  F40.2X  F41.0  F41.1  F41.8  F41.9  F42*  F93.0 | F32.X  F33.X  F34.1 | F91.1  F91.2  F91.9  F91.3 |

*Obsessive-Compulsive Disorder – no longer classified as an anxiety disorder (but was at the time of the MacArthur trial, thus allowing comparisons)
